# KLICK Syndrome Linked to a *POMP* Mutation Has Features Suggestive of an Autoinflammatory Keratinization Disease

**DOI:** 10.3389/fimmu.2020.00641

**Published:** 2020-04-30

**Authors:** Takuya Takeichi, Masashi Akiyama

**Affiliations:** Department of Dermatology, Nagoya University Graduate School of Medicine, Nagoya, Japan

**Keywords:** autoinflammatory keratinization diseases, keratosis linearis with ichthyosis congenita and sclerosing keratoderma syndrome, inflammation, proteasome maturation protein, unfolded protein response

## Abstract

Keratosis linearis with ichthyosis congenita and sclerosing keratoderma (KLICK) syndrome is a rare autosomal recessive skin disorder characterized by palmoplantar keratoderma, linear hyperkeratotic plaques, ichthyosiform scaling, circular constrictions around the fingers, and numerous papules distributed linearly in the arm folds and on the wrists. Histologically, the affected skin shows hypertrophy and hyperplasia of the spinous, granular, and horny epidermal layers with mild infiltration of inflammatory cells in the upper dermis. There are 14 patients with KLICK syndrome described in the literature, and they all carry the same nucleotide deletion. Proteasome maturation protein (POMP), encoded by *POMP*, is an ubiquitously expressed protein that functions as a chaperone for proteasome maturation. KLICK syndrome is caused by a reduction in POMP levels that leads to proteasome insufficiency in differentiating keratinocytes. It is noteworthy that *POMP* is also known to be the causative gene for proteasome-associated autoinflammatory syndrome-2 (PRAAS2). It is considered that the disrupted proteasome assembly caused by the *POMP* mutation might lead to both skin inflammation and then hyperkeratosis in KLICK syndrome. Inflammation caused by the hyperactivation of innate immunity occasionally leads to inflammatory diseases of the skin, recently denoted as autoinflammatory keratinization diseases (AiKDs). We propose that KLICK syndrome caused by the specific 1-bp nucleotide deletion mutation in the regulatory region of *POMP* might be in a spectrum of proteasome-associated phenotypes.

## Introduction

Keratosis linearis with ichthyosis congenita and sclerosing keratoderma (KLICK) syndrome (MIM 601952) is an autosomal recessive skin disorder characterized by palmoplantar keratoderma, linear hyperkeratotic plaques, ichthyosiform scaling, circular constrictions around the fingers, and numerous papules distributed linearly in the arm folds and on the wrists (1, 2). KLICK syndrome is a rare disease, with only several pedigrees having been described. In 2010, a single nucleotide deletion in the 5′ untranslated region (UTR) of *POMP* (rs112368783) was identified in 12 KLICK patients (3). All described patients with KLICK syndrome harbored the same homozygous 1-bp deletion in the 5′ UTR of the *POMP* gene (3–5).

Autoinflammatory keratinization disease (AiKD) is an umbrella term recently introduced to describe inflammatory keratinization diseases caused by mutations in single genes associated with autoinflammatory diseases (6, 7). AiKDs are genetically heterogeneous, and their different subtypes show various clinical features, complications, and prognoses (8–11). We propose that KLICK syndrome associated with the *POMP* mutation be categorized as an AiKD.

## What is Klick Syndrome?

In 1989, Pujol RM et al. reported four members of a consanguineous family presenting a disorder similar to KLICK syndrome (12). They described a congenital syndrome consisting of (i) generalized ichthyosiform dermatosis, (ii) diffuse palmoplantar keratoderma with sclerosis, deformities, pseudoainhum, and functional impairment, (iii) multiple keratotic papules in a symmetrical linear cordlike arrangement involving the flexures and exhibiting peculiar acrosyringial keratoses, (iv) a possible autosomal recessive pattern of inheritance, (v) inconsistent dental abnormalities, and (vi) the absence of systemic involvement (e.g., neurological or ophthalmological) (12). Their peculiar clinical pictures were described as “congenital ichthyosiform dermatosis with linear keratotic flexural papules and sclerosing palmoplantar keratoderma” (12). A biopsy specimen from an area with ichthyosiform dermatosis showed irregular hyperplasia, hypergranulosis, hyperkeratosis, and parakeratosis (12). In addition, the dermis showed mild superficial perivascular lymphohistiocytic infiltrates. In 1997, Vahlquist et al. reported an additional case and proposed the acronym KLICK to define this uncommon disorder (2, 13). Using a combination of homozygosity mapping and candidate gene screening, Dahlqvist J et al. identified a single-nucleotide deletion in the 5′ UTR of *POMP* that was identified in 12 KLICK patients (2, 12, 14, 15). The families were nonrelated and originated from Spain, Italy, Netherlands, Sweden, and Norway (3). Haplotype analysis using microsatellite markers flanking *POMP* in the eight affected probands found at least five different haplotypes, suggesting that the c.-95delC variant is a recurrent, rather than a founder, mutation (3).

Recently, an unusual case of KLICK syndrome was reported whose initial clinical diagnosis was erythrokeratoderma or loricrin keratoderma (5). The patient had diffuse thin white scaling skin and well-demarcated nonmigratory symmetrical erythematous and hyperkeratotic plaques on the limbs and extremities (5). A skin biopsy revealed irregular acanthosis and hypergranulosis associated with numerous enlarged keratohyaline granules (5). The presence of well-demarcated erythematous and hyperkeratotic plaques, as seen in erythrokeratoderma, is not a clinical feature that has been commonly reported for KLICK syndrome (5). To date, ~20 cases of KLICK syndrome associated with the recurrent hotspot mutation in the 5′ UTR of *POMP* have been reported. Some cases of KLICK syndrome show significant improvement of the skin eruptions with etretinate therapy (4, 5, 13).

## Klick Syndrome and Proteasome Insufficiency

POMP, encoded by *POMP*, is an ubiquitously expressed protein that functions as a chaperone for proteasome maturation of the standard proteasome and the immunoproteasome (3, 16). Constitutive proteasomes and immunoproteasomes shape the peptide repertoire presented by major histocompatibility complex class I (MHC-I) molecules by harboring different sets of catalytically active subunits and plays a critical role in homeostasis and immunity (17). The ubiquitin–proteasome system (UPS) is a selective proteolytic system in which substrates are recognized and tagged with ubiquitin for processive degradation by the proteasome (18). Cells rapidly shift to immunoproteasome formation in response to proinflammatory cytokines produced by the innate immune system early upon infection (19). POMP is strongly and consistently expressed from the basal to the granular layer of the epidermis in sections from healthy subjects, whereas in KLICK patients, the staining is strong in the basal layer with a gradual decrease toward the granular layer (3). Thus, KLICK syndrome is caused by a reduction in POMP levels that leads to proteasome insufficiency in differentiating keratinocytes (20). POMP functions as a chaperone for proteasome assembly and interacts with an initially formed α ring for subsequent sequential incorporation of β subunits into both the standard multiprotein complex 20S proteasome and the immunoproteasome (16). Proteasome inhibition is known to cause increased endoplasmic reticulum (ER) stress (21). CCAAT/enhancer-binding protein homologous protein (CHOP) is a transcription factor induced by persistently elevated ER stress and by the unfolded protein response (UPR). Immunostaining of CHOP shows a gradual, but moderate, increase from the spinous to the granular layer in the normal skin. In contrast, the staining of CHOP is clearly increased in the granular layer, and weak staining is also seen in the horny layer in KLICK skin (3). This staining pattern is consistent with the abnormal distribution of POMP and the proteasome subunits, α7 and β5, in the granular layer of individuals with KLICK (3).

Dahlqvist et al. revealed that the knockdown of POMP expression in cell cultures results in decreased amounts of proteasome subunits (20). Additionally, *POMP* knockdown causes a slight increase in the ER chaperone BiP in keratinocyte-derived HaCaT, an immortalized human keratinocyte cell line, cells but not in HeLa cells, supporting the idea of tissue-specific sensitivity to ER stress (20). ER stress is activated by impairment in the degradation of misfolded proteins due to dysfunctional proteasomes (22). Importantly, physiological ER stress is required for the maintenance of normal biological functions in skin, including keratinocyte differentiation, a vital process in competent skin barrier formation (23). Activation of the UPR likely leads to the disturbance in terminal differentiation of keratinocytes (22). The ER stress that leads to UPR in differentiating cells is very mild and occurs at levels that do not cause keratinocytes apoptosis, whereas severe ER stress, i.e., that which exceeds mild ER stress, does cause epidermal apoptosis (22). Excessive ER stress has been reported to be involved in the pathogenesis of certain skin disorders, including inflammatory skin diseases (i.e., psoriasis, rosacea, vitiligo, etc.) (23).

## Pomp-Related Systemic Autoinflammatory Diseases

It is noteworthy that *POMP* is also known to be a causative gene for proteasome-associated autoinflammatory syndrome (PRAAS2) (24), also known as POMP-related autoinflammation and immune dysregulation disease (PRAID) (16). In fact, a homozygous single-nucleotide deletion in the 5′ UTR of *POMP* causes KLICK syndrome, whereas heterozygous frameshift variants in the penultimate exon of *POMP* result in systemic autoinflammatory diseases (Table 1) (16, 24). Autosomal recessive homozygous or compound heterozygous loss-of-function mutations in *PSMB8*, which encodes the inducible proteasome component β5i, cause a syndrome that has historically been referred to as joint contractures, muscle atrophy, microcytic anemia, and panniculitis-induced childhood-onset lipodystrophy syndrome (25), Nakajo–Nishimura syndrome (26, 27), or chronic atypical neutrophilic dermatosis with lipodystrophy and elevated temperature (CANDLE) (28). These conditions form a single disease spectrum of PRAAS (24). Brehm et al. reported additional disease-causing variants in three proteasome genes, *PSMA3* encoding α7, *PSMB4* encoding β7, *PSMB9* encoding β1i, and *POMP*, and also established the digenic inheritance of PRAAS (24). Additive depletion of two proteasome subunits by small-interfering RNA (siRNA) was shown to cause more severe assembly defects and decreases in proteolytic function than monogenic inherited PRAAS (24). Recently, Sarrabay et al. described a patient exhibiting a PRAAS phenotype due to a homozygous mutation in *PSMB10*, which led to interferon (IFN) type I dysregulation, PSMB10 maturation defect, and enzymatic impairment (29). Notably, among these mutations, the 5′ UTR of *PSMB4* variant (c.-9G>A) reduced the expression levels of the mutant transcripts (24). Patients with PRAID show a unique constellation of early-onset combined immunodeficiency, inflammatory neutrophilic dermatosis, and autoimmunity (16). Although the proposed mechanism of diseases is different (haploinsufficiency vs. dominant-negative effect), the clinical inflammatory phenotype of PRAID is similar to that of PRAAS. PRAID has been considered as the proteasome-associated autoinflammatory syndrome-2 (PRAAS2, MIM 618048).

**Table 1:** Comparison of two syndromes associated with *POMP* mutations.

**Disease (references)**	**KLICK syndrome (2, 3, 12)**	**PRAAS2/PRAID (16, 24)**
*POMP* mutations	c.-95delC (5′ UTR)	c.334_335delAT (p.Ile112Trpfs^*^3), c.342_348delinsACC (p.Phe114Leufs^*^18), c.344_345insTTTGA (p.Glu115Aspfs^*^20)
Skin manifestations	Palmoplantar keratoderma, linear hyperkeratotic plaques, ichthyosiform scaling	Perplexing constellation of papulo-erythematous skin lesions on the face, trunk, and extremities; panniculitis, necrotizing lesions, and scarring
Histological characteristics	Irregular hyperplasia, hypergranulosis, superficial hyperkeratosis parakeratosis; nonspecific/lymphohistiocytic infiltrates of inflammatory cells in the upper dermis	Neutrophilic infiltration and leukocytoclastic vasculitis consistent with neutrophilic dermatosis
Extracutaneous features		Early-onset combined immunodeficiency and autoimmunity

*KLICK, keratosis linearis with ichthyosis congenita and sclerosing keratoderma; POMP, proteasome maturation protein; PRAAS2, proteasome-associated autoinflammatory syndrome-2; PRAID, POMP-related autoinflammation and immune dysregulation disease; UTR, untranslated region*.

The first reported patient with PRAAS2 had a heterozygous frameshift mutation c.344_345insTTTGA (p.Glu115Aspfs^*^20) in exon 5 of *POMP* (24). This *POMP* mutation likely causes haploinsufficiency, as supported by previous findings in which an ~50% reduction in POMP levels is sufficient to cause impaired proteasome activity and cell death *in vitro* (30, 31). Although the detailed cutaneous features of PRAAS2 were lacking, this patient with *POMP* mutation presented with periorbital erythema, annular plaques, and acanthosis nigricans (24). Subsequently, Poli et al. detected two *de novo* frameshift *POMP* mutations, c.334_335delAT (p.Ile112Trpfs^*^3) and c.342_348delinsACC (p.Phe114Leufs^*^18), in two unrelated affected individuals with PRAID. Both heterozygous frameshift mutations in the penultimate exon of *POMP* escape nonsense-mediated messenger RNA (mRNA) decay and result in a truncated protein that perturbs proteasome assembly by a dominant-negative mechanism (16). The aggregation of ubiquitin-modified proteins that resulted from the proteasome dysfunction was observed in fibroblast cell lines from these two patients (16). The cutaneous manifestations of PRAID are a perplexing constellation of papulo-erythematous skin lesions on the face, trunk, and extremities, and these eruptions can progress to necrotizing lesions and subsequent scarring (16). Skin biopsies from the affected lesions in both PRAID patients revealed neutrophilic infiltration and leukocytoclastic vasculitis consistent with neutrophilic dermatosis (16). These clinical and histological findings suggest more severe inflammation in the skin eruptions of PRAID than in those of KLICK syndrome. HEK293T cells transfected with the mutant *POMP* constructs detected in the patients with PRAID showed increased expression of genes that are induced by type 1 interferon, indicating a disease-promoting toxic dominant-negative effect of the truncated protein and resembling the findings in patients with PRAID (16).

## Autoinflammatory Diseases Caused by Inflammasome Hyperactivation

The KLICK syndrome phenotype, which is limited to the epidermis, is reminiscent of another group of diseases that are caused by mutations in the pyrin/leucine-rich repeat (LRR) domains of *NLRP1*, mutations that lead to multiple self-healing palmoplantar carcinoma (MSPC)/familial keratosis lichenoides chronica (FKLC); also, the PRAID phenotype is reminiscent of diseases that are caused by mutations in the *NLRP1* gene causative of NLRP1-associated autoinflammation with arthritis and dyskeratosis (NAIAD)/juvenile-onset recurrent respiratory papillomatosis (JRRP) (32). Characteristic clinical features of FKLC include tiny papules on the trunk and extremities that become confluent, resulting in linear and reticulate patterns, and seborrheic dermatitis-like eruptions on the face (32). The lesions have a chronic and often progressive course. Excessive activation of inflammasomes has been demonstrated in patient keratinocytes, and inflammasome-dependent interleukin (IL)-1 cytokines have been shown to cause inflammatory FKLC (32). We have classified FKLC as an original member of the AiKDs (6). Thereafter, one family with an *NLRP1* mutation between the NACHT and LRR domains and one sporadic patient with an *NLRP1* mutation in the FIIND domain were reported to have autoinflammation symptoms, including follicular keratosis in the skin, as well as polyarthritis (NAIAD), and those symptoms were responsive to IL-1 inhibition (33) (Supplementary Table 1). Serum IL-18 and caspase-1 levels were elevated in the patients with NAIAD, which suggests the hyperactivation of the NLRP1 inflammasome (33). Moreover, very recently, a homozygous *NLRP1* gain-of-function mutation located between the NACHT and LRR domains in siblings with a syndromic form of JRRP was reported (34). JRRP is a rare and debilitating childhood disease that presents with recurrent growth of papillomas in the upper airway (34). Drutman et al. revealed that patient-derived keratinocytes secreted elevated levels of IL-1β at baseline, and both patients displayed elevated levels of inflammasome-induced cytokines in the serum (34). Notably, these patients had unique cutaneous eruptions: keratosis pilaris on the legs and lower trunk, palmoplantar wart-like hyperkeratotic papules, and atrophoderma vermiculata on the cheeks (34). Thus, several distinct phenotypes resulting from the different mutations in *NLRP1* have been reported. Although phenotype/genotype correlations are still unclear, the pathogenesis of these skin inflammatory diseases is linked to gain of function in the NLRP1 inflammasome.

## Other Cutaneous Phenotypes Associated With Autoinflammation in the Skin

Recently, we proposed that porokeratosis be categorized as an AiKD (8). Porokeratosis is a genetically heterogeneous disorder that can be caused by mutations in any of the four genes involved in the mevalonate pathway (*MVK, MVD, PMVK*, and *FDPS*) or by mutations in *SLC17A9* (8, 35). Interestingly, one of the major causative genes of porokeratosis, *MVK*, is also known to be causative of a conventional autoinflammatory disease, hyperimmunoglobulinemia D, and periodic fever syndrome (MIM 260920) (8). In 2010, mutations in genes encoding γ-secretase subunits (*NCSTN, PSENEN*, and *PSEN1*) were reported in patients presenting with hidradenitis suppurativa (HS) (36). The essential subunit of the γ-secretase complex, an endoprotease complex, catalyzes the intramembrane cleavage of integral membrane proteins, such as Notch receptors, and amyloid-beta precursor protein (37). γ-Secretase deficiency could also regulate inflammation by processing important cytokine receptors such as IL-1β R1/R2 and IL-6R (37). HS caused by mutations in genes encoding γ-secretase subunits was suggested to have features characteristic of an AiKD (9, 38). Mutations involving different autoinflammatory genes (*MEFV, NLRP3, NLRP12, NOD2, LPIN2*, and *PSTPIP1*) have been reported in syndromic HS [pyoderma gangrenosum, acne, and hidradenitis suppurativa (PASH) syndrome] as well as in pyoderma gangrenosum (PG), a prototypic neutrophilic dermatosis (39). Marzano et al. reported that increase in skin expression of IL-1β and IL-17 and the presence of mutations in these genes involved in autoinflammation indicate that PG is a polygenic autoinflammatory condition, as previously demonstrated in PASH (39, 40).

Some studies of mouse models of autoinflammatory diseases mimicking neutrophilic dermatosis in humans were also reported. Neutrophilic dermatosis encompasses disorders that are characterized by neutrophilic infiltration with ulceration in the upper dermis not associated with infection, such as Sweet's syndrome and PG (41). Mutations in the *PTPN6* gene that encodes the protein tyrosine phosphatase Src homology region 2 (SH2) domain-containing phosphatase 1 (SHP-1) have been linked with autoinflammatory and autoimmune diseases in humans (41). Hypomorphic *Ptpn6* mutant mice with a homozygous Tyr208Asn amino acid alteration mutation (exhibiting spontaneous inflammation or spin) develop persistent footpad swelling and suppurative inflammation that are very similar to neutrophilic dermatosis in humans (42–44).

## Klick Syndrome Has Features Characteristic of an Aikd

A number of unique features support KLICK syndrome as an AiKD (6, 7). First, the skin of affected individuals histologically shows hypertrophy and hyperplasia of the spinous, granular, and horny epidermal layers (2, 5, 12). In addition, mild, spare nonspecific/lymphohistiocytic infiltrates of inflammatory cells are seen in the upper dermis (2, 12). These histological features suggest that the primary and main inflammation sites of KLICK are the epidermis and the upper dermis. Second, the ichthyosis, the palmoplantar keratoderma with constricting bands, and the keratotic papules that are seen in KLICK syndrome are usual and common eruptions caused by hyperkeratosis, which is the main characteristic phenotype of AiKDs. Finally, the causal genetic variant of KLICK is consistent with proteasome dysfunction (3, 20). Although the molecular mechanism of the disease is unclear, KLICK patients exhibit increased expression of the ER stress markers BiP and/or CHOP, and prolonged ER stress is known to induce inflammation as well as to be responsible for the pathogenesis of numerous chronic inflammatory diseases (16, 20). Although there are no reports of hyperactivation of innate immunity or of the induction of a type 1 interferon response in KLICK patients, the chronic ER stress present in the epidermis of KLICK patients may eventually trigger autoinflammation (16).

**Figure 1:**
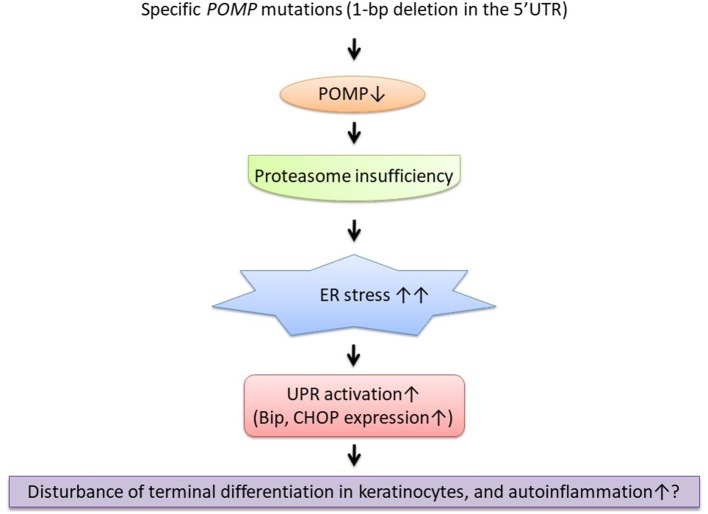
The suggestive pathogenesis of keratosis linearis with ichthyosis congenita and sclerosing keratoderma (KLICK) syndrome as an autoinflammatory keratinization disease (AiKD). ER, endoplasmic reticulum; POMP, proteasome maturation protein; UPR, unfolded protein response.

Like porokeratosis and hidradenitis suppurativa, as novel genetic causes and predisposing factors for inflammatory keratinization disorders have been revealed, we assume that more skin inflammatory diseases will be categorized as AiKDs. In KLICK syndrome, the disrupted proteasome assembly caused by the *POMP* mutation leads to hyperkeratosis (22) and might also lead to autoinflammation by increased type 1 interferon signaling (Figure 1). Recently, significant upregulation of IL-17/tumor necrosis factor alpha (TNF-α)-related genes has been reported in autosomal recessive congenital ichthyosis patients (45, 46). Furthermore, clinically, anti-inflammatory therapies (e.g., anti-TNF-α antibodies, anti-IL-17 antibodies) have been reported as useful treatments for inherited ichthyoses and in other AiKDs (7, 47, 48). Therefore, because KLICK syndrome is also a form of inherited ichthyosis (49), anti-inflammatory therapies might be useful for KLICK patients (48).

## Discussion

This review summarized the pathogenesis and clinical features of KLICK syndrome associated with a specific *POMP* mutation. Some KLICK cases show improvement of the skin eruptions with etretinate, one of the systemic retinoids, therapy. Although oral retinoids have been described as efficient therapeutic agents for severe ichthyotic disorders, those treatments have various severe side effects. Moderate to severe ichthyosis is known to have a significant impact on quality of life (50). Thus, we strongly hope that safe, effective therapies will be established for patients with KLICK syndrome in the near future based on an understanding of the molecular pathogenesis of KLICK syndrome.

Both the clinical eruptions and the histological findings described in the reported KLICK patients clearly show mild to moderate inflammation in the affected skin (2, 5, 12). As we have cited that prolonged ER stress is known to induce inflammation in numerous chronic inflammatory skin diseases (23), the inflammation seen in KLICK syndrome could be considered to result from continuous abnormal ER stress in keratinocytes. Hetz et al. notes that under “irremediable ER stress,” the UPR actively promotes proteotoxicity, sterile inflammation, and apoptosis (51). Agyemang et al. report that Mendelian defects in the proteasome cause protein accumulation, which can trigger interferon-dependent autoinflammatory disease (52). However, in the literature, there are little data revealing the detailed inflammatory pathways from ER stress and UPR activation in KLICK syndrome. Although there are no therapeutic interventions available that target proteasome assembly, improving the proteostatic potential of cells by intervention with UPR (51) might be a therapeutic strategy for KLICK syndrome. To reduce patient distress, further clinical and laboratory investigations for the diagnosis and treatment of KLICK are needed.

## Author Contributions

TT and MA contributed conception and design of the study and read and approved the submitted version. TT wrote the first draft of the manuscript. MA contributed to manuscript revision.

## Conflict of Interest

The authors declare that the research was conducted in the absence of any commercial or financial relationships that could be construed as a potential conflict of interest.

## Abbreviations

AiKDs: autoinflammatory keratinization diseases
ER: endoplasmic reticulum
HS: hidradenitis suppurativa
KLICK: keratosis linearis with ichthyosis congenita and sclerosing keratoderma
PG: pyoderma gangrenosum
POMP: proteasome maturation protein
PRAAS2: proteasome-associated autoinflammatory syndrome
PRAID: POMP-related autoinflammation and immune dysregulation disease
UPR: unfolded protein response
UTR: untranslated region.
