# Supplementary material for: KLICK Syndrome Linked to a POMP Mutation Has Features Suggestive of an Autoinflammatory Keratinization Disease
Source: Front Immunol. 2020 Apr 30;11:641. doi: 10.3389/fimmu.2020.00641 (PMC7203212; doi:10.3389/fimmu.2020.00641)
Supplement: Supplementary file 1 [file Table_1.docx]

**Supplementary Table S1. Comparison of reported phenotypes associated with *NLRP1* mutations**

| **Disease** | **MSPC** | **FKLC** | **NAIAD** | **JRRP** |
| --- | --- | --- | --- | --- |
| ***NLRP1* mutations** | p.Ala54Thr, p.Ala66Val, p.Met77Thr | p.Phe787_Arg843del (in frame deletion of exon 5) | c.2176C>T (p.Arg726Trp), c.3641C>G (p.Pro1214Arg) | c.2819C>A (p.Thr755Asn) |
| **Location of mutations** | Pyrin domain | LRR domain | Between the NACHT and LRR domains, and in the FIIND domain | Between the NACHT and LRR domains |
| **Skin manifesta-tions** | ulcerative, hyperkeratotic nodular growths on plantar and palmar skin; keratoacanthoma; well-differentiated squamous cell carcinomas; irregular and thickened nails; hyperkeratosis pilaris | multiple discrete and semi-confluent lichenoid papules on the arms, legs, and lower trunk; plantar keratosis; follicular hyperkeratosis; macular amyloidosis; thickened fragmented nail plates with surrounding inflammation involving the nail folds and the distal margin | disseminated follicular hyperkeratosis, dyskeratosis | atrophoderma vermiculata on the cheeks, plantar warts, keratosis pilaris on the buttocks and thighs |
| **Histological characteris-tics** | circumscribed acanthosis; hyperkeratosis; dyskeratosis; papillomatosis with focal lichenoid infiltrates | mild acanthosis and hyperkeratosis; colloid bodies (apoptotic keratinocytes) in the papillary dermis | acanthosis with scattered to confluent dyskeratotic keratinocytes; dyskeratotic cells in all parts of the epidermal layer except the basal layers | cutaneous histological features not described |
| **Extracuta-neous features** | squamous dyskeratotic lobules |  | recurrent fever, recurrent elevated CRP, chronic infection (*Giardia intestinalis*, candidiasis), polyarticular arthritis, subglottic oedema, uveitis, photophobia, corneal dyskeratosis and neovascularisa-tion dyskeratosis, vitamin A deficiency | recurrent respiratory papillomatosis |

Abbreviations: FIIND, function to find domain; FKLC, familial keratosis lichenoides chronica; JRRP, juvenile-onset recurrent respiratory papillomatosis; LRR, leucine-rich repeat; MSPC, multiple self-healing palmoplantar carcinoma; NACHT, neuronal apoptosis inhibitor protein, major histocompatibility complex class II transcription activator, incompatibility locus protein from *Podospora anserine* and telomerase-associated protein; NAIAD, NLRP1-associated auto-inflammation with arthritis and dyskeratosis
